# Tumor classification and biomarker discovery based on the 5’isomiR expression level

**DOI:** 10.1186/s12885-019-5340-y

**Published:** 2019-02-07

**Authors:** Shengqin Wang, Zhihong Zheng, Peichao Chen, Mingjiang Wu

**Affiliations:** 1 College of Life and Environmental Science, Wenzhou University, Wenzhou, 325035 China; 2 Translational Medicine Research Institute, Zhejiang University, Hangzhou, China

**Keywords:** Tumor classification, isomiR, Genetic algorithm, Random forest, miRNA

## Abstract

**Background:**

The miRNA isoforms (isomiRs) have been suggested to regulate the same pathways as the canonical miRNA and play an important biological role in miRNA-mediated gene regulation. Recently, a study has demonstrated that the presence or absence of all isomiRs could efficiently discriminate amongst 32 TCGA cancer types. Besides, an effective reduction of distinguishing isomiR features for multiclass tumor discrimination must have a major impact on our understanding of the disease and treatment of cancer.

**Methods:**

In this study, we have constructed a combination of the genetic algorithms (GA) with Random Forest (RF) algorithms to detect reliable sets of cancer-associated 5’isomiRs from TCGA isomiR expression data for multiclass tumor classification.

**Results:**

We obtained 100 sets of the optimal predictive features, each of which comprised of 50–5’isomiRs that could effectively classify with an average sensitivity of 92% samples from 32 different tumor types. We calculated the frequency with which a 5’isomiR found in these sets as measuring its importance for tumor classification. Many highly frequent 5’isomiRs with different 5′ loci from canonical miRNAs were detected in these sets, supporting that the isomiRs play a significant role in the multiclass tumor classification. The further functional enrichment analysis showed that the target genes of the 10 most frequently appearing 5’isomiRs were involved in the activity of transcription activator and protein kinase and cell-cell adhesion.

**Conclusions:**

The findings of the present study indicated that the 5’isomiRs might be employed for multiclass tumor classification and the suggested that GA/RF model could perform effective tumor classification by a series of largely independent optimal predictor 5′ isomiR sets.

## Background

The accumulation of genetic alteration drives cancer development and progression [1]. The Cancer Genome Atlas (TCGA) consortium integrated comprehensive clinicopathologic annotation data together with molecular profiles of over 11,000 human tumors across 30 different human tumor types [2]. Analyzing these large datasets can provide more exciting opportunities to better understand the tumor characteristics and discover novel and effective predictive and prognostic tumor biomarkers and therapeutic targets. While most of the previous studies on tumor classifications have focused primarily on the gene expression data, including RNA-seq and microarray data [3–5]. miRNAs and isoforms of human miRNAs (isomiRs) also play essential roles and may serve as potential biomarkers for tumor classification [6–11]. The isomiRs are predominantly generated from the alternative cleavage of Drosha or Dicer and 3′addition events, which produce mature miRNA different from the canonical miRNA by a few nucleotides at the 5′ or 3′ end and designated as 5’isomiR or 3’isomiR [12, 13]. Both computational and experimental analyses revealed that the isomiRs are involved in regulating distinctive target genes and could play a crucial biological role in miRNA-mediated gene regulation [14–18]. In a recently conducted study, the presence or absence of 7466 isomiRs could be effectively discriminated amongst 32 TCGA cancer types with 90% sensitivity [19]. Moreover, an average sensitivity of 82% was achieved by using 456 most significant isomiRs. In the present study, we aimed to evaluate the effective reduction of discriminant isomiR features for multiclass TCGA tumor discrimination classification.

The filter, wrapper and embedded methods are typically utilized for feature selection, though all of them are not good at dealing with data which contain a large number of collinear variables. [20, 21]. The isomiRs may belong to the same miRNA family, the same miRNA cluster, or some of them even have same seed region, leading to similar or related function and highly correlated expression. In the previous studies, the wrapper method could outperform embedded methods by the combined machine learning algorithm for classification [21]. The genetic algorithms combined with the machine learning algorithm, which employs a GA as the search engine for feature subset selection and the machine learning algorithm as the classification tool, was efficiently used for classification of gene expression data [3, 4]. This algorithm could identify and classify more than 90% of samples from 31 tumor types with a set of 20 genes [3]. Beside the machine learning algorithms -- such as support vector machines (SVM), sparse representation (SR), sparse representation classifier (SRC), random forest (RF), and k-Nearest Neighbors (KNN) -- has been extensively applied in cancer prognosis and prediction analysis [3, 22–25]. Nevertheless, GA has proven ability to detect the optimal classifier effectively for multiclass cancer discrimination [4, 26]. GA is based on Darwin’s theory of natural evolution, and it is typically implemented using computer simulations in which an optimization problem is specified. GA is frequently used to generate high-quality solutions to optimization problems using genetic operators: selection, crossover, and mutation [27]. In this study, we constructed a combination of the GA with random forest algorithms to detect reliable sets of cancer-associated 5’isomiRs from TCGA isomiR expression data.

Furthermore, 5’isomiR may target very different transcripts as compared with their canonical miRNAs attributed to shifting in the seed region (typically 2–7 nt of the miRNA), which is recognized to be very critical in determining miRNA target specificity [28–31]. Various 5′ isomiRs play an important role in suppression and progression of cancer [32, 33]. Using the combined GA/RF algorithms, reliable sets of candidate tumor biomarkers for multiclass tumor discrimination was detected by combining all the miRNA isoforms with same loci of 5′ end together in TCGA isomiR expression data. In this step, the miRNA isoforms with same loci of 5′ end will be left with only one in the reliable sets, which will dramatically reduce the type of isomiRs. The findings of the present study revealed that the 5’isomiRs might be utilized for effective tumor classification and classifier can achieve an average sensitivity of 91.5% with only 50–5’isomiRs.

## Methods

### Datasets

All the TCGA isomiR expression data for 10,999 TCGA datasets representing 33 tumor types was downloaded (April 2018) from the TCGA data portal (https://tcga-data.nci.nih.gov). Only primary solid tumor samples with infix ‘01’ in the TCGA sample barcode, with the exception of the blood samples derived from acute myeloid leukemia (LAML; sample infix ‘03’) were included in the study. After excluding all samples that were annotated as ‘potentially problematic’ datasets (file_annotations.txt files), 9085 eligible datasets (isoform.quantification.txt files) were finally included for further analysis (Table 1). For each dataset, we generated 5’isomiR profiles by combing all the sequences together with same loci at 5′ end, and the expression level of each 5’isomiR was calculated by the sum of all corresponding RPM (read per million) values. In order to avoid noise generated due to poorly expressed isomiRs, we only included the 5’isomiR profiles with read depths of ≥10 in more than 10 samples. Next, we log2-transformed the combined normalized read depths for each 5’isomiR; however, as the read depths ≤1 RPM were considered noise, we filtered them by assigning all values less than 1 the value 1 before log transformation.

**Table 1:** Tumor types and number of TCGA isomiR samples used in the analysis

Tumor Types	# of samples
Adrenocortical carcinoma [ACC]	79
Bladder Urothelial Carcinoma [BLCA]	366
Breast invasive carcinoma [BRCA]	1064
Cervical squamous cell carcinoma and endocervical adenocarcinoma [CESC]	299
Cholangiocarcinoma [CHOL]	35
Colon adenocarcinoma [COAD]	386
Lymphoid Neoplasm Diffuse Large B-cell Lymphoma [DLBC]	46
Esophageal carcinoma [ESCA]	183
Head and Neck squamous cell carcinoma [HNSC]	487
Kidney Chromophobe [KICH]	60
Kidney renal clear cell carcinoma [KIRC]	455
Kidney renal papillary cell carcinoma [KIRP]	261
Acute Myeloid Leukemia [LAML]	105
Brain Lower Grade Glioma [LGG]	497
Liver hepatocellular carcinoma [LIHC]	356
Lung adenocarcinoma [LUAD]	445
Lung squamous cell carcinoma [LUSC]	419
Mesothelioma [MESO]	82
Ovarian serous cystadenocarcinoma [OV]	349
Pancreatic adenocarcinoma [PAAD]	152
Pheochromocytoma and Paraganglioma [PCPG]	178
Prostate adenocarcinoma [PRAD]	472
Rectum adenocarcinoma [READ]	144
Sarcoma [SARC]	243
Skin Cutaneous Melanoma [SKCM]	94
Stomach adenocarcinoma [STAD]	425
Testicular Germ Cell Tumors [TGCT]	149
Thyroid carcinoma [THCA]	483
Thymoma [THYM]	122
Uterine Corpus Endometrial Carcinoma [UCEC]	514
Uterine Carcinosarcoma [UCS]	55
Uveal Melanoma [UVM]	80

### Multiclass GA/RF classifier

In this study, we used the GA/RF based model for tumor classification. GA/RF utilizes a GA to select a set of salient features from input and classification module using RF [27]. The selected features were used as inputs to RF [22]. In a genetic algorithm, a population of strings (designated as chromosomes), which encode candidate solutions (the 5’isomiR signature in this case) to an optimization problem, evolves toward better solutions. The evolution typically starts from a population of randomly generated 5’isomiR sets and occurs in generations. In the present study, the parameters including the “chromosome” length, the “population” size and the maximum number of “generations” were set to 50 (including 50–5’isomiR set), 50, and 300, respectively. For RF classification, the *randomForest* was used (https://cran.r-project.org/web/packages/randomForest/randomForest.pdf). Besides, we use SVM classification for comparison, and the *e1071* package in R with linear kernel function was run (https://cran.r-project.org/web/packages/e1071/index.html).

For tumor, classification prediction may vary based on different samples assigned to the training set. We repeated the above the GA/RF procedure100 times. During each of the 100 runs, the training and testing were carried out, each time using one distinct subset of randomly selected for training and the remaining subsets for testing. In a given run, the training sets were generated by randomly selecting 75% of each cancer’s available tumor datasets, and the testing sets were generated by the remaining 25% datasets. Finally, we achieved optimal 5’isomiR sets after 300 generations of GA/RF steps.

### 5’isomiR target prediction and function enrichment analysis

Using the TargetScanHuman (http://www.targetscan.org) and the TargetScanHuman Custom (http://www.targetscan.org/vert_50/seedmatch.html) prediction of the target genes of 5’isomiRs with original seed region along with different seed region of canonical miRNA, respectively, were performed [40]. Then, the predicted target genes were submitted to the functional annotation tools of DAVID for the functional enrichment analysis [41, 42]. For functional annotation, the 3 Gene Ontology items (GOTERM_BP_FA GOTERM_CC_FAT, and GOTERM_MF_FAT) were selected with the Enrichment Thresholds or EASE set as 0.001.

## Results

### Tumor classification

**Fig. 1:**
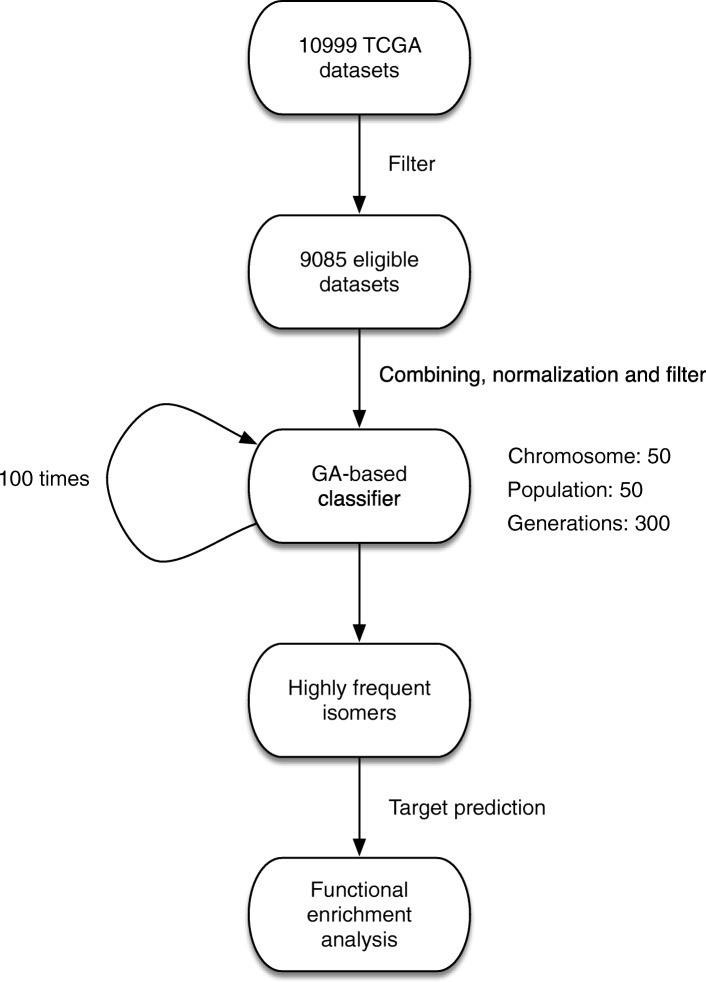
The work flow of our GA/RF based algorithm for detecting reliable sets of cancer-associated 5’isomiRs from TCGA isomiR expression data

**Fig. 2:**
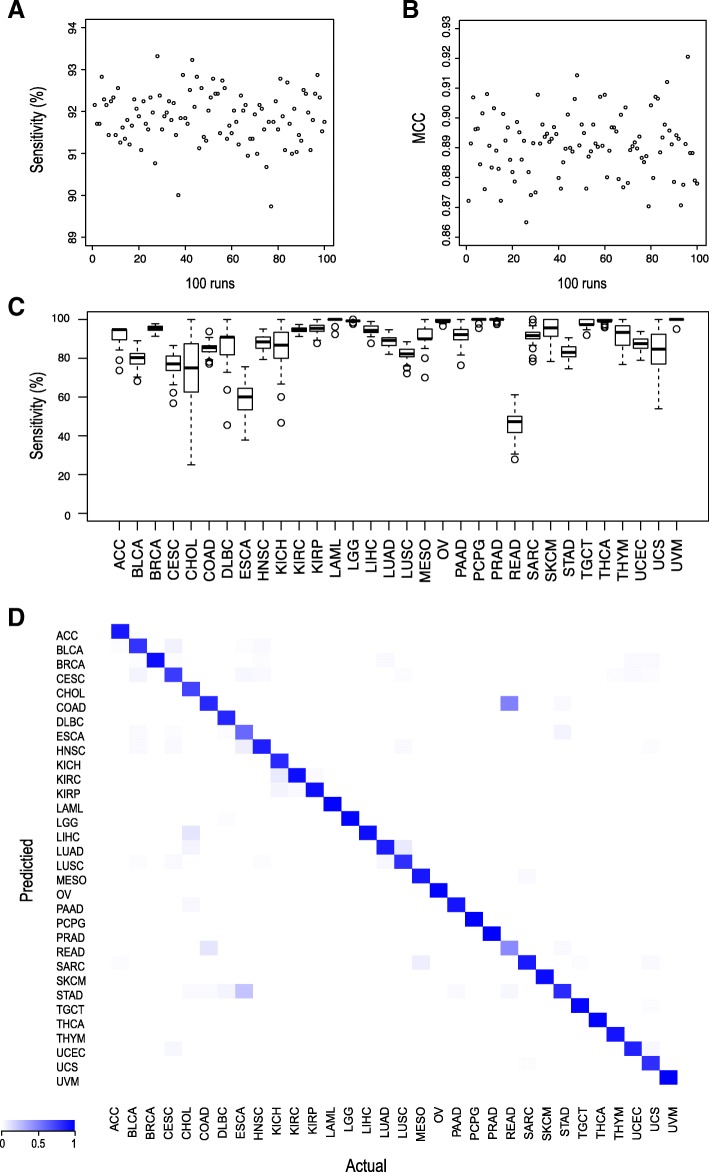
Analysis of GA/SVM-derived optimal feature sets for 100 runs generated by GA/SVM. **a** The average sensitivity for 100 generated predictor sets. **b** The average MCC (Matthew’s Correlation Coefficient) for 100 generated predictor sets [43]. **c** The prediction accuracies for 32 tumor classifications. **d** The average sensitivity of test-set samples predicted to be each of the 32 tumor types. X-axis and Y-axis list the actual and the predicted cancer type, respectively. The color of each cell in the heatmap is the average sensitivity of the test-set samples originally as the cancer type in X-axis to be predicted as the cancer type in Y-axis

Here, we have constructed a combination of the genetic algorithms (GA) with Random Forest (RF) algorithms to detect reliable sets of cancer-associated 5’isomiRs from TCGA isomiR expression data for multiclass tumor classification (Fig. 1). After 100 independent runs, the prediction accuracies of each classifier for each cancer could be obtained with 300 generations of GA. Based on the initial pre-selected set population size, we obtained 100 sets of the optimal predictive features, each of which is comprised of 50–5’isomiRs. The GA/RF and GA/SVM achieved quite similar results (the average sensitivities were 92 and 91.5%, respectively), and then our following analysis only used the result from GA/RF classifier. The 100 generated predictor sets required relatively similar classification accuracies(Fig. 2a, Fig. 2b), which indicated that our selected 5’isomiR sets were remarkably accurate for multiclass tumor classifications. Besides, the prediction accuracies for cholangiocarcinoma (CHOL), rectum adenocarcinoma (READ) and esophageal carcinoma (ESCA), were recorded to be relatively low, indicating that these tumors were often classified as other types (Fig. 2c). Interestingly, the samples of these cancers could be effectively classified in some runs by altering the training and test set, with different isomiR sets, except for READ. Further, in order to investigate which tumor types could be hardly distinguished from all others, we calculated the mean prediction sensitivity for all runs. Notably, similar tumor classification was obtained as reported previously (Fig. 2d). Moreover, the majority of samples from READ tumor were misclassified as colon adenocarcinoma (COAD), which could be attributed to similar molecular expression, histology, and anatomical location [19, 34]. These findings suggested that the GA/RF model could perform effective tumor classification by a series of largely independent optimal predictor 5′ isomiR sets.

### Characterization of the highly frequent 5’isomiRs

For all 100 runs, we acquired 100 sets of GA/RF classifiers and each set comprised of 50 items. Then, the frequency of selected 5′ isomiR across all the optimal predictive set was calculated. The relative significance of each 5′ isomiR for tumor classification was assessed by counting how often it appeared in these predicted optimal feature sets. For the functional enrichment analysis, we included all 5′ isomiRs with the frequency of ≥11 in these sets. It is difficult to randomly select a gene for more than 11 times in a 100 subset from 100 runs for a given dataset of 2231 samples, and the significance of adjusted *p*-value was calculated as less than 0.01 followed by Bonferroni correlation for multiple testing. Finally, 41 highly frequent 5’isomiRs were selected (Table 2). Notably, of which 28 had different 5′ loci than that of the canonical miRNA from miRBase 21, and some 5’isomiRs even originated from the same pre-miRNA.

**Table 2:** Detail description of 41 highly frequent 5′ isomiRs in 100 generated predictor sets

5’isomiR ID	Chromosome	Strand	Start site	Corresponding miRNA ID	Frequency in 100 runs	Canonical seed region?
313	Chr1	+	209,432,166	hsa-miR-205-5p	88	Y
698	Chr6	+	52,144,401	hsa-mir-206	85	Y
233	Chr7	–	27,169,550	hsa-mir-196b-5p	75	N
121	Chr2	+	176,150,330	hsa-miR-10b-5p	40	N
39	Chr2	+	176,150,329	hsa-miR-10b-5p	36	Y
151	Chr17	–	48,579,926	hsa-miR-10a-5p	32	Y
301	Chr2	–	219,001,669	hsa-miR-375	24	Y
193	Chr2	–	219,001,670	hsa-miR-375	24	N
317	Chr2	–	219,001,671	hsa-miR-375	23	N
103	Chr3	+	189,829,974	hsa-mir-944	21	N
350	Chr2	+	176,150,331	hsa-miR-10b-5p	20	N
529	Chr1	–	220,117,937	hsa-miR-215-5p	18	N
275	Chr17	–	48,579,925	hsa-miR-10a-5p	18	N
506	Chr3	+	189,829,975	hsa-mir-944	16	Y
207	Chr5	–	149,062,395	hsa-miR-584-5p	16	N
188	Chr12	+	6,963,742	hsa-miR-200c-3p	15	Y
594	Chr12	+	62,603,694	hsa-let-7i-5p	14	N
119	Chr11	–	64,891,426	hsa-miR-194-5p	14	N
884	ChrX	+	136,550,892	hsa-miR-934	13	Y
297	ChrX	–	151,958,652	hsa-miR-224-5p	13	N
264	Chr17	–	1,713,934	hsa-miR-22-3p	13	N
208	Chr17	–	48,579,928	hsa-miR-10a-5p	13	N
91	Chr6	–	71,403,576	hsa-miR-30a-3p	12	N
90	Chr17	+	31,560,016	hsa-miR-193a-5p	12	Y
854	ChrX	+	136,550,928	hsa-mir-934	12	N
449	Chr11	–	64,891,390	hsa-miR-194-3p	12	N
372	Chr14	–	101,560,347	hsa-miR-1247-3p	12	N
247	Chr1	–	207,802,474	hsa-miR-29b-3p	12	N
124	Chr5	+	149,428,977	hsa-miR-143-3p	12	N
120	Chr1	–	220,118,228	hsa-miR-194-5p	12	N
572	Chr20	+	62,564,971	hsa-miR-133a-3p	11	N
475	Chr2	+	176,150,328	hsa-miR-10b-5p	11	N
429	Chr20	+	62,554,351	hsa-miR-1-3p	11	Y
392	Chr9	–	21,512,179	hsa-miR-31-5p	11	N
37	Chr6	–	71,403,617	hsa-miR-30a-5p	11	N
358	Chr7	–	129,774,987	hsa-miR-183-5p	11	N
324	Chr7	–	129,770,466	hsa-miR-182-5p	11	N
316	Chr1	+	1,167,124	hsa-miR-200b-5p	11	Y
248	Chr7	–	130,877,491	hsa-miR-29b-3p	11	N
232	Chr1	+	1,167,160	hsa-miR-200b-3p	11	Y
113	Chr21	+	16,539,101	hsa-miR-99a-5p	11	Y

**Fig. 3:**
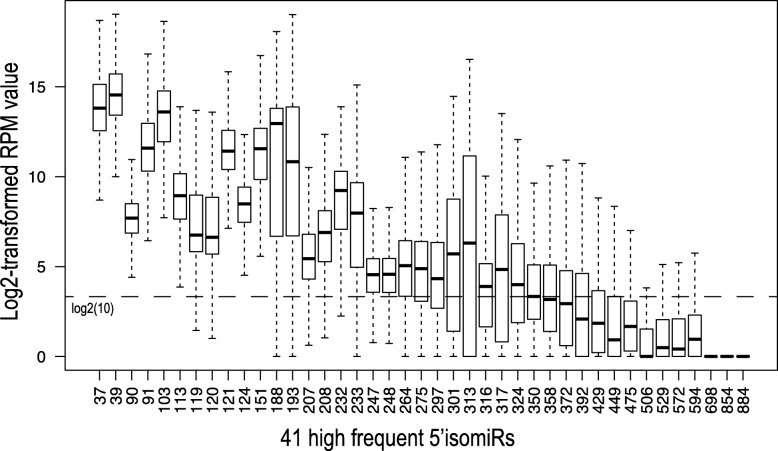
The expression level of 41 highly frequent isomiRs in 100 generated predictor sets. X-axis lists the 5’isomiR ID used in this study (Detail description can be found in Table 2). Y-axis is the log2-transformed RPM value. The line indicates the value of log2-transformed 10 rpm. The outliers are hidden

Next, we examined the expression level of these highly frequent isomiRs (Fig. 3). Many of them showed higher than 10 rpm, which was the threshold value as derived from a previous study for presence or absence of isomiRs [19]. Noticeably, some of them showed extremely lower expression, nearly all of 5’isomiRs showed less than 10 rpm. It is worth noting that we combined 5′ isomiRs with same 5′ loci together in this study.

### Tumor classification and functional analysis with the 9 most frequently appearing 5′ isomiRs

**Fig. 4:**
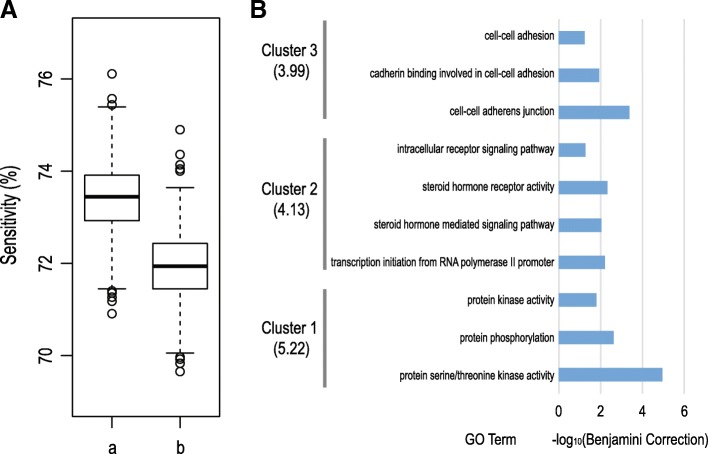
Tumor classification and functional enrichment analysis with the 9 most frequently appearing 5’isomiRs. **a** Tumor classification. Y-axis is the average sensitivity for 1000 randomly produced test sets. “a” is the 9 most frequently appearing 5’isomiRs. In the list of 9 most frequently appearing 5’isomiRs, two 5’isomiRs were from hsa-miR-10b-5p and three 5’isomiRs belonged to has-375. “b” is obtained from “a” by removing three 5’isomiRs which had different 5′ loci from the canonical miRNA (one was from hsa-miR-10b-5p and two belonged to has-375). **b** Bar plot shows the enriched GO terms from DAVID functional annotation analysis. The clusters integrated with enrichment score are shown as Y-axis. The –log10(*P*-value after correlation) is plotted on the X-axis

Next, the 9 most frequently appearing 5’isomiRs from all 100 runs were selected to determine whether a rational classification could be obtained using a reduced set. The training set and the corresponding test set were randomly produced 1000 times. Then, the RF algorithm was performed to determine the prediction accuracy of multiclass tumor classification (Fig. 4a). We could still achieve a reasonable prediction accuracy (the average sensitivity was 73.7%). In the list of 9 most frequently appearing 5’isomiRs, two 5’isomiRs were from hsa-miR-10b-5p and three 5’isomiRs belonged to hsa-miR-375. In order to investigate whether these 5’isomiRs with different 5′ loci could provide an additional contribution to the tumor classification, we removed the three 5’isomiRs which had different 5′ loci from the canonical miRNA, and randomly rebuilt the training set and the corresponding test set 1000 times again. Finally, we could only obtain an average sensitivity of 72.3% by the RF algorithm, which suggested that the isomiR shown different contribution in multiclass tumor classification than the canonical miRNA.

Furthermore, to investigate the candidate target genes of the 9 most frequently appearing 5’isomiRs, the TargetScanHuman was performed for the 5–5’isomiRs with canonical seed region and the TargetScan Custom was used for other 4–5’isomiRs with a shift in seed regions. The target genes predicted were subjected to the functional enrichment analysis. Finally, 2345 genes were recognized by the DAVID web tools to analyze the functional annotation and the detection of enriched functional categories. The gene ontology enrichment analysis suggested that these target genes were highly enriched in genes implicated in the activity of transcription activator and protein kinase and cell-cell adhesion (Fig. 4b).

## Discussion

We report a novel GA/RF analytical model for multiclass tumor classification using the miRNA expression data that may reveal effective predictive and prognostic biomarkers and therapeutic targets for drug development. With an average sensitivity of 92%, we were able to accurately classify the tumor samples using 100 different 50–5’isomiR sets, though some 5’isomiRs appeared repetitively in the sets. These predictive 5’isomiRs sets could achieve similar prediction accuracies with slight overlap; suggesting that even less sensitive 5’isomiRs could be detected for the tumor classification. Notably, most of the tumor types could be easily distinguished with high sensitivity. However, there were also some cancers that exhibited low prediction accuracy due to similar histology and anatomical location [19, 34]. In this study, we used GA algorithm to obtain the optimal isomiR set for maximizing the prediction accuracy in all TCGA cancer types but not for some individual cancer. Therefore, the samples from some cancers that cannot be classified by one set may be successfully classified by another set. In addition, we calculated the frequency with which each 5’isomiRs appeared in these sets. More than half of 41 highly frequent 5′ isomiRs showed different 5′ loci than the canonical miRNA, supporting that the isomiRs play a significant role in the multiclass tumor classification. It is noted that our analysis only included tumor samples, and we cannot distinguish cancer-specific isomiRs from tissue-specific biomarkers. Actually, a group from Saarland university had utilized a tissue specificity index to define the distribution of miRNA across 61 tissue biopsies of two individuals, and people can check whether the detected isomiRs correspond to the tissue-specific miRNA expression in their web-based repository [35].

In a recent study, the RNA-seq expression data analysis revealed that many development-related genes are essential for the analysis of TCGA cancer classification [3]. Similar clues were also revealed in the present study. 5’isomiR-233, one of the most frequently appearing 5’isomiRs in 100 generated predictor sets, derived from the shift in the seed region of canonical hsa-mir-196b-5p, which usually appears to be expressed from the intragenic regions of HOX gene clusters that are major regulators of animal development [36]. Increasing studies have suggested that 5’isomiR-313, combined from the isomiRs with identical 5′ loci of the canonical hsa-miR-205-5p, play an important role in normal cellular development as well as in cancer development [37, 38]. Moreover, TBX5, one of the most important genes for tumor classification from the previous study [3], could be regulated by one of the 5 most frequently appearing 5’isomiRs in our sets (miR-10b-5p/5’isomiR-39) as derived from the TargetScanHuman prediction.

Using only 50–5’isomiRs, the present GA/RF model could achieve comparable prediction performances consistent with previous report, with an average accuracy of 90% for all isomiRs [19]. We also detected the similar discriminatory isomiRs as their finding. For example, the isomiRs of has-miR-205-5p and has-miR-944, two of the most important miRNAs detected by the method using the presence or absence of isomiRs amongst 32 TCGA cancer types, are also listed in the ten highly frequent isomiRs from 100 generated predictor sets. The isomiR of hsa-mir-196b-5p, the most frequently appearing 5’isomiRs with a shift in seed regions found in our study, showed a high VI score in previous report [19]. Further, we reduced the number of features by employing two strategies. In the first approach, we combined the isomiR with same 5′ loci to reduce the type of isomiRs. While in the second approach, the GA-based isomiR selection reduced the feature selection significantly. We also found that the 9 most frequently appearing 5’isomiRs could achieve an average sensitivity of 73.7%, suggesting that a reasonable accurate performance could be obtained with less number of features. The features can be further reduced by additional approaches, including hybrid GA-based machine learning method [39]. The highly expressed 5’isomiRs (rpm > 10 in all samples) and slightly expressed 5’isomiRs (rpm < 10 nearly in all samples), demonstrated that the expression level of isomiRs could also be beneficial for the tumor classification.

## Conclusions

In conclusion, the present study demonstrated that the 5’isomiRs might be employed for multiclass tumor classification and the suggested that GA/RF model could perform effective tumor classification by a series of largely independent optimal predictor 5′ isomiR sets.

## Abbreviations

AML: Acute myeloid leukemia
CHOL: Cholangiocarcinoma
COAD: Colon adenocarcinoma
ESCA: Esophageal carcinoma
GA: Genetic algorithms
isomiR: MiRNA isoform
MCC: Matthew’s Correlation Coefficient
READ: Rectum adenocarcinoma
RF: Random forest
RPM: Read per million
SVM: Support vector machine
TCGA: The Cancer Genome Atlas
